# Degeneration of olivospinal tract in the upper cervical spinal cord of multiple system atrophy patients: Reappraisal of Helweg's triangular tract

**DOI:** 10.1111/bpa.13226

**Published:** 2023-11-16

**Authors:** Takashi Ando, Yuichi Riku, Akio Akagi, Hiroaki Miyahara, Takashi Uematsu, Ikuko Aiba, Jun Sone, Masahisa Katsuno, Mari Yoshida, Yasushi Iwasaki

**Affiliations:** ^1^ Department of Neurology Japanese Red Cross Aichi Medical Center Nagoya Daiichi Hospital Nagoya Aichi Japan; ^2^ Department of Neuropathology Institute for Medical Science of Aging, Aichi Medical University Nagakute Aichi Japan; ^3^ Department of Neurology Nagoya University Graduate School of Medicine Nagoya Aichi Japan; ^4^ Department of Neurology National Hospital Organization Higashinagoya National Hospital Nagoya Aichi Japan; ^5^ Department of Clinical Research Education Nagoya University Graduate School of medicine Nagoya Aichi Japan

**Keywords:** alpha‐synuclein, autopsy, Helweg's triangular tract, multiple system atrophy, spinal cord, tract degeneration

## Abstract

Multiple system atrophy (MSA) is an adult‐onset neurodegenerative disorder that presents with variable combinations of autonomic dysfunction, cerebellar ataxia, parkinsonism, and pyramidal signs. The inferior olivary nucleus is targeted in MSA, with a phenotype of olivopontocerebellar atrophy in particular, and involvement of the olivocerebellar tract is well known. However, degeneration of the olivospinal tract has not been studied in MSA. We examined 97 spinal cords from consecutively autopsied patients with MSA. Myelin staining revealed that 22 cords (22.7%) had small, bilateral, triangular‐shaped tract degeneration in the boundary of the anterior and lateral funiculi, which appeared continuously from C1 to C5. The anatomical pathway of the degenerated tract was consistent with the description of the olivospinal tract provided by Helweg in 1888. The MSA patients showing degeneration of this tract were younger at disease onset (average: 56.4 ± 8.7 years, range: 42–74), and had longer disease duration (average: 10.1 ± 4.8 years, range: 2–25) and more severe olivopontocerebellar changes compared to other MSA patients. Quantitative analyses revealed that patients with olivospinal tract degeneration had a lower neuronal density in the inferior olivary nucleus compared to other patients. Microglial density in this tract was negatively correlated with the neuronal density in the inferior olivary nucleus. The densities of glial cytoplasmic inclusions in the inferior olivary nucleus and in the olivospinal tract were strongly correlated with each other. Neurologically healthy controls (*n* = 22) and disease controls with Lewy body disease (*n* = 30), amyotrophic lateral sclerosis (*n* = 30), and progressive supranuclear palsy (*n* = 30) did not present the olivospinal tract degeneration. Our results indicate an impairment of the neural connection between the inferior olivary nucleus and the spinal cord in MSA patients, which may develop in a descending manner.

## Graphical abstract

We observed olivospinal tract degeneration in autopsied MSA patients. The tract degeneration was closely linked to neuronal loss in the inferior olivary nucleus and an olivopontocerebellar atrophy phenotype associated with long clinical duration. Although the physiological impact of this change remains unclear, an impairment of the neural connection between the inferior olivary nucleus and spinal cord is implied.

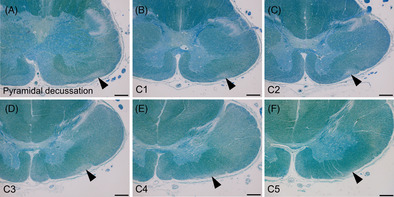

## 1 INTRODUCTION

Multiple system atrophy (MSA) is an adult‐onset neurodegenerative disorder that presents with variable combinations of autonomic dysfunction, cerebellar ataxia, parkinsonism, and pyramidal signs [1, 2]. The presence of alpha‐synuclein‐immunoreactive glial cytoplasmic inclusions (GCIs), associated with prominent neuronal loss and astrogliosis in the striatonigral (SN) or olivopontocerebellar (OPC) systems, constitutes the neuropathological hallmark of MSA [3, 4].

Another feature of MSA is pathological change in the spinal cord [5]. For example, neuronal loss in the intermediolateral column and Onuf's nucleus is an important finding of MSA and forms the basis of autonomic dysfunction [2, 5]. Moreover, alpha‐synuclein‐immunopositive GCIs and neuronal inclusions are observed in the spinal cord [5]. Degeneration of the anterior and lateral corticospinal tracts is also observed in the spinal cords of patients with MSA [6, 7, 8], which may be a pathological background for pyramidal signs. However, tract degeneration other than that in the corticospinal tracts has not yet been reported in the spinal cords of patients with MSA.

In this study, we assessed 97 spinal cords from consecutively autopsied MSA patients. We identified 22 patients with bilateral triangular‐shaped tract degeneration in the boundary between the anterior and lateral funiculi, which continuously appeared across the upper cervical segments. The anatomical pathway of the degenerated tract was consistent with the olivospinal tract originally described by H. Helweg in 1888 [9]. Degeneration of this tract has not been reported in any other disorder, and the existence of the descending olivospinal tract described by Helweg has been the subject of some controversy [10, 11, 12]. This study aims to clarify the relationship between olivospinal tract degeneration and pathological changes of the inferior olivary nucleus by assessing clinical and neuropathological findings from autopsied MSA patients. Moreover, disease‐specificity of the olivospinal tract degeneration was addressed among neurodegenerative disorders.

## 2 MATERIALS AND METHODS

### 2.1 Patients with MSA

Written informed consent was obtained from the patients' relatives before autopsy. All studies in humans were approved by the research ethics committee of Aichi Medical University. We reviewed the clinical and pathological findings of 201 patients with neuropathologically established MSA who were consecutively autopsied between 1978 and 2020 at the Institute for Medical Science of Aging, Aichi Medical University. The clinical diagnosis of MSA was made by neurologists from Nagoya University, Aichi Medical University, or affiliated hospitals. The clinical information was obtained from medical records and clinicopathological conferences of neurological experts. All autopsied patients were of East Asian origin. We excluded 104 of the 201 patients with MSA due to unavailability of the upper cervical cord between C1 and C4 (*n* = 74), insufficient clinical information (*n* = 13), artifacts in the upper cervical cord (*n* = 2), and comorbid pathological changes in the central nervous system (CNS) (severe ischemic change, *n* = 13; intracerebral hemorrhage, *n* = 1; encephalitis, *n* = 1). Finally, 97 patients were included in the study.

### 2.2 Clinical findings

Clinical data were retrospectively acquired from medical records. Disease onset was defined as awareness of either motor or autonomic symptoms, as mentioned in the current MSA criteria [13]. Disease duration was defined as the number of years from disease onset to death or induction of artificial ventilation, as described previously [14]. The clinical phenotypes were classified as MSA‐cerebellar type (MSA‐C) or MSA‐parkinsonian type (MSA‐P), depending upon the predominant motor signs and symptoms [13].

### 2.3 Tissue preparation and immunohistochemical procedures

Tissues were fixed in 20% formalin for at least 2 weeks [15], and the right hemisphere was cryopreserved. We obtained 8‐mm sections of the left hemisphere along the coronal plane using a brain knife and a standardized slicer. The brainstem was sectioned in the transverse plane, and the cerebellum was cut sagittally to obtain 5‐mm thick sections. The medulla oblongata was cut at 5 mm caudal from the obex. The regions of interest were then trimmed for embedding after macroscopic observation [16]. Sections of 9‐μm thickness were prepared for hematoxylin–eosin (H&E) and Klüver‐Barrera (KB) staining and silver impregnation with the Gallyas method, while 4.5‐μm thick sections were prepared for immunohistochemistry. The following primary antibodies were used for immunohistochemical analysis: anti‐alpha‐synuclein (polyclonal rabbit, 1:1000; Sigma–Aldrich, St. Louis, MO), anti‐phosphorylated alpha‐synuclein (pSyn#64; mouse monoclonal, 1:6000; Wako Pure Chemical Industries, Osaka, Japan), anti‐phosphorylated neurofilament (clone SMI31; mouse monoclonal, 1:4000; Covance, Berkeley, CA), anti‐beta‐amyloid (clone 12B2; mouse monoclonal, 1:1000; IBL, Gunma, Japan), anti‐phosphorylated tau (clone AT‐8; mouse monoclonal, 1:4000; Innogenetics, Ghent, Belgium), anti‐Iba1 (rabbit polyclonal, 1:500; Wako Pure Chemical Industries), anti‐CD‐163 (mouse monoclonal, 1:500; Wako Pure Chemical Industries), and anti‐polyglutamine (clone 5TF1‐1C2; monoclonal mouse, 1:3000; Sigma–Aldrich). Using the standard avidin‐biotin method, secondary immunolabeling was performed using biotinylated immunoglobulins, and 3,3′‐diaminobenzidine (Wako Pure Chemical Industries) was used as the chromogen.

### 2.4 Identification of the spinal cord segment

We identified the spinal cord segments using the classical method [17]. Briefly, the anterior and posterior roots were thicker and larger in the C4 to Th1 segments (cervical enlargement); however, they became smaller at the level of the Th2 segment and onwards. In the lower cord, the L1 to S1 roots (lumbar enlargement) were thicker and larger; however, these became conspicuously thinner from the S2 level. Therefore, we prepared transverse slices of the spinal cord at the point where the nerve root of each segment began most rostrally. All available spinal cord segments were trimmed for embedding.

### 2.5 Semi‐quantitative assessment of the neuropathological findings

Two neurologists (T.A. and M.Y.) conducted the neuropathological assessments. The neuropathological diagnosis of MSA was made on the basis of the widespread presence and abundance of GCIs labeled using alpha‐synuclein‐immunostaining and Gallyas staining [3, 13]. Additionally, the semi‐quantification of neuronal loss and astrogliosis in each anatomical region was conducted using H&E staining and graded as follows: grade 0, normal appearance; grade 1+, definite astrogliosis in the absence of definite neuronal loss; and grade 2+, apparent neuronal loss and astrogliosis. We also classified the severity of olivopontocerebellar atrophy (OPCA) and striatonigral degeneration (SND) into four grades (0: no changes; I: mild changes; II: moderate changes; and III: severe changes) using Jellinger's neuropathological MSA scale [18]. Since it is not always possible to confidently discriminate between true Lewy bodies and Lewy body‐like neuronal inclusions in MSA using anti‐alpha‐synuclein immunohistochemistry, the presence of concomitant Lewy bodies in the substantia nigra, locus coeruleus, or dorsal nucleus of the vagus was evaluated using H&E staining [5, 19]. We also assessed the presence of concomitant AT8‐immunopositive neurofibrillary tangles (NFT) [20] and beta‐amyloid deposition [21].

### 2.6 Definition of tract degeneration in the spinal cord

We evaluated the spinal cord tracts using myelin staining with the KB method. Tract degeneration was defined as the loss of myelin sheaths, which systematically and continuously appeared across segments. We excluded the loss of myelin staining in a single segment or that with fuzzy edges from the definition of tract degeneration because ischemic changes, focal inflammation, or artifacts, rather than systematic tract degeneration, were possible in these settings.

### 2.7 Quantitative analyses

Quantitative assays were performed in 10 MSA patients with olivospinal tract degeneration and in 10 MSA patients without it. Disease durations and Jellinger's pathological scores for OPCA and SND were matched between the two groups. To assess neuron densities in the inferior olivary nucleus, we performed immunohistochemistry in slides of medulla oblongata using an anti‐NeuN antibody with DAB labeling, followed by counterstaining with luxol fast blue (LFB). This allowed us to identify neurons in the inferior olivary nucleus. The photomicrograph of whole, bilateral inferior olivary nuclei was acquired with a digital camera under ×100 magnification, followed by the unification of captured visual fields into a single panel. The photomicrographs were processed using the ‘color‐deconvolution’ plug‐in of Image‐J software (NIH, MD), and the DAB and LFB channels were separately acquired. Each channel was binarized via the ‘Otsu threshold’ in the same settings. Neuronal density was calculated as [DAB‐labeled particle counts/area (mm^2^) of inferior olivary nucleus]. The neuronal densities were obtained from both sides of inferior olivary nucleus and averaged to obtain a single outcome for each patient. We also assessed microglial and GCI densities in the olivospinal tract in the 10 MSA patients with olivospinal tract degeneration. immunohistochemistry with anti‐Iba1 or anti‐alpha‐synuclein were performed in the C2 segments, followed by LFB counterstaining. The photomicrographs underwent color‐deconvolution, and microglial density was calculated as [DAB‐labeled area (pixels)/area (pixels) of olivospinal tract] in both sides and later averaged (Figure S1).

### 2.8 Age and sex‐matched controls and disease controls

We also examined the spinal cords of 20 age‐ and sex‐matched controls without neurodegenerative disorders who were consecutively autopsied between 2008 and 2020. Moreover, patients with Lewy body disease (LBD) (*n* = 30; underwent autopsy between 2015 and 2020), amyotrophic lateral sclerosis (ALS) (*n* = 30; underwent autopsy between 2016 and 2020), progressive supranuclear palsy (PSP) (*n* = 30; underwent autopsy between 2012 and 2020), spinocerebellar ataxia type 1 (SCA1) (*n* = 6; underwent autopsy between 1997 and 2021), and spinocerebellar ataxia type 2 (SCA2) (*n* = 2; underwent autopsy between 1997 and 2018) were included to assess the disease‐specificity of the tract degeneration.

### 2.9 Statistical analysis

Data are presented as means with standard deviation (SD) and range values. The Mann–Whitney *U* test was used to compare continuous and ordinal variables, while Fisher's exact test was used to compare categorical variables. Furthermore, the Kruskal–Wallis test was used to compare continuous and ordinal variables among three or more groups. Statistical significance was set at a *p*‐value of 0.05, and all statistical analyses were performed using the EZR (Saitama Medical Center, Jichi Medical University, Saitama, Japan) package of R and R commander (The R Foundation for Statistical Computing, Vienna, Austria) [22].

## 3 RESULTS

### 3.1 Olivospinal tract degeneration was specifically observed in MSA patients

**FIGURE 1:**
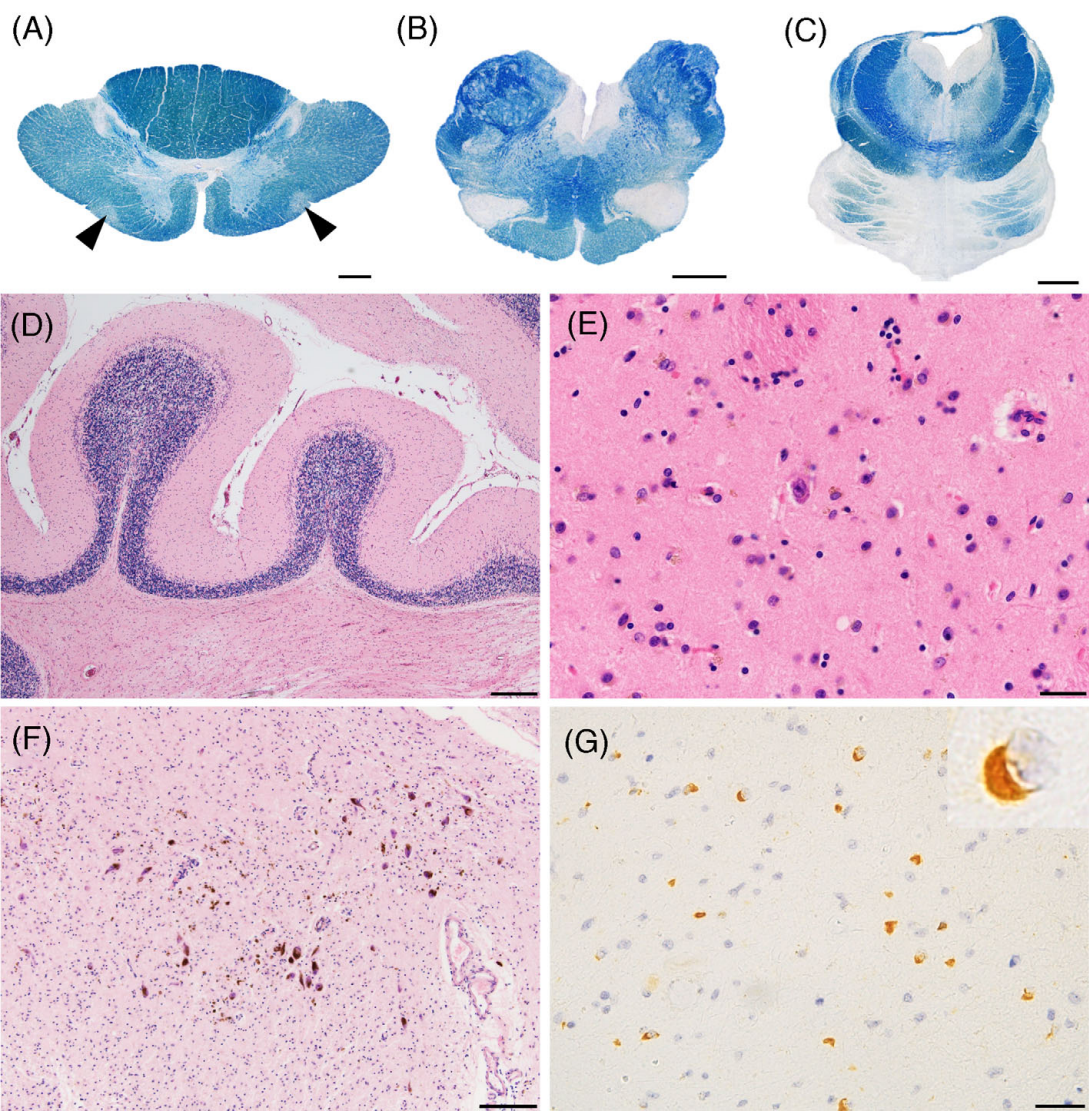
Neuropathologic findings in an MSA patient with olivospinal tract degeneration. (A–D) Olivospinal tract degeneration (A, arrowheads) is observed at the C2 level of the spinal cord, and was associated with neuron loss and astrogliosis in the olivo‐pontine‐cerebellar system. The inferior olivary nucleus (B) and pontine nucleus (C) are markedly atrophied, and neuronal loss, Bergmann's astrogliosis in the Purkinje cell layer and rarefaction of the white matter are observed in the cerebellum (D). (E, F) The extent of neuronal loss in the putamen (E) and substantia nigra (F) is less than that observed in the olivo‐pontine‐cerebellar system. (G) The inferior olivary nucleus displays alpha‐synuclein‐immunopositive glial cytoplasmic inclusions. A–I: Patient 14. Scale bars: A, 500 μm; B–C, 2 mm; D, 200 μm; E, G, 20 μm; F, 100 μm; A–C: Klüver‐Barrera staining, D–F: hematoxylin–eosin staining, G: anti‐alpha‐synuclein immunohistochemistry. MSA, multiple system atrophy.

**FIGURE 2:**
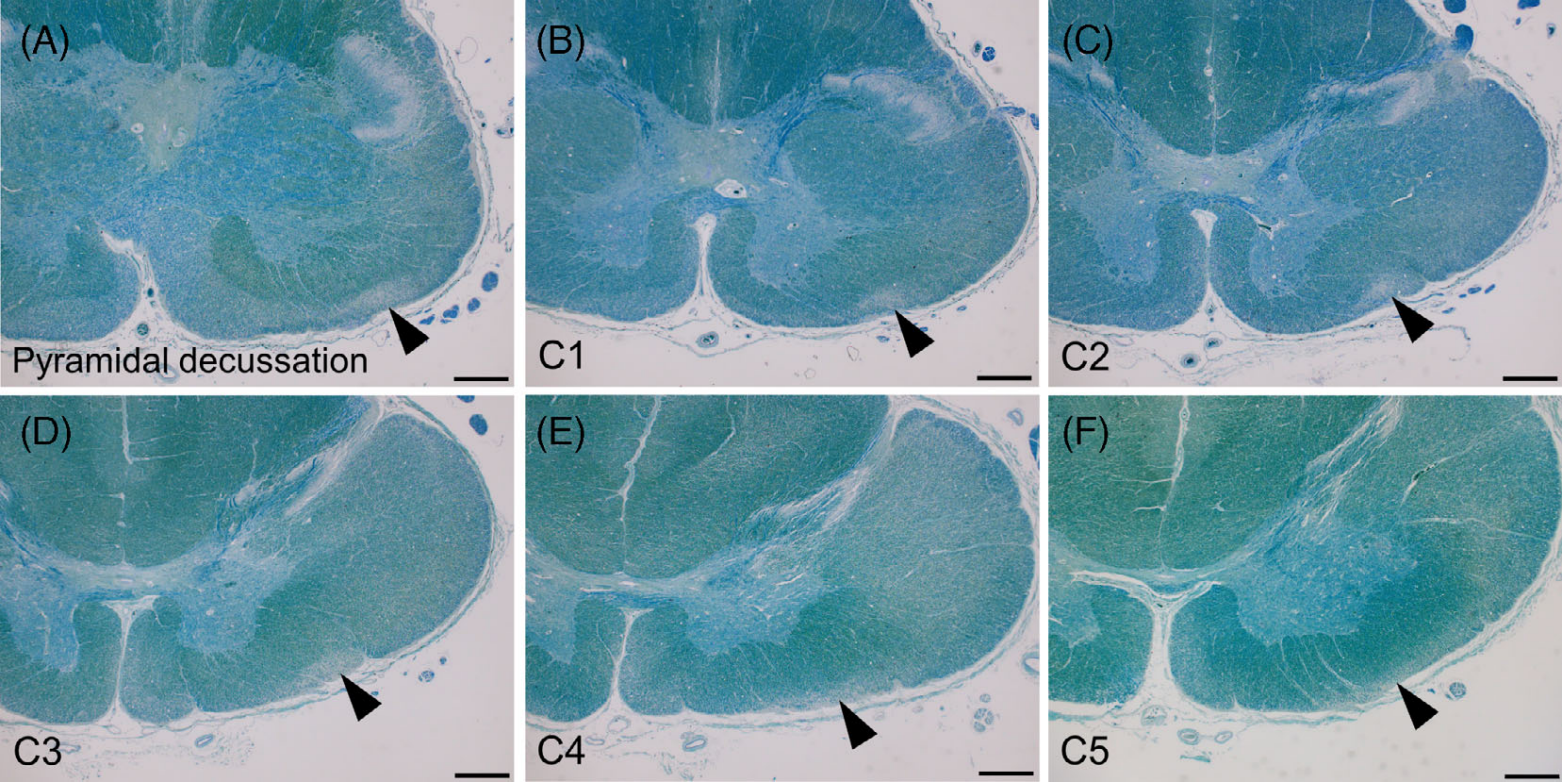
Observation of olivospinal tract degeneration in MSA across multiple slices. (A–F) Multiple sections from the inferior portion of the medulla oblongata and upper spinal cord segments exhibit tract degeneration in the boundary of the anterior and lateral funiculi (arrowheads). A–I: Patient 12. Scale bars: A–F, 500 μm. A–F: Klüver‐Barrera staining. MSA, multiple system atrophy.

**FIGURE 3:**
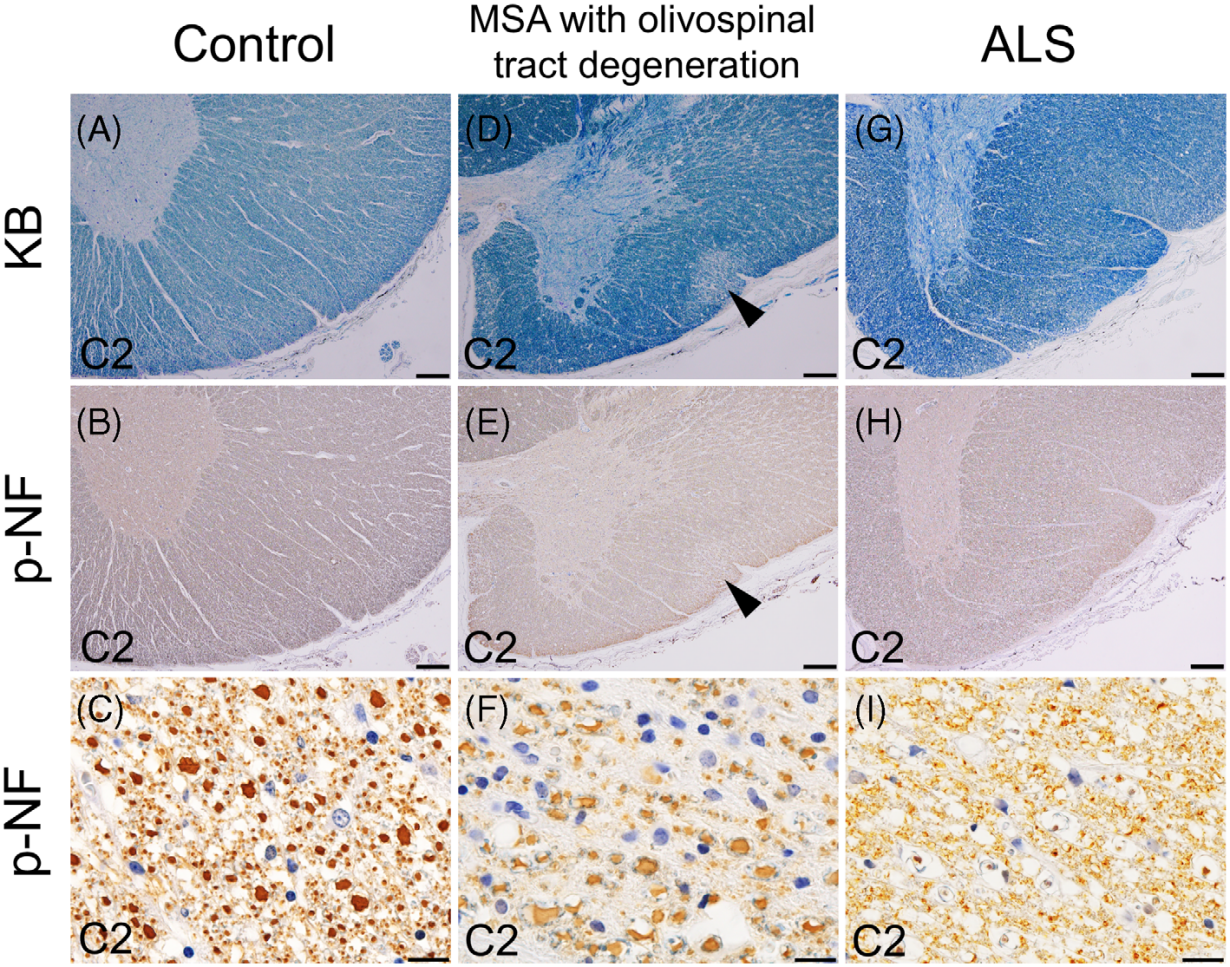
Axonal changes in the olivospinal tract in MSA and other conditions. The panels display the C2 segment of the cervical cord. (A–C) Anti‐phosphorylated neurofilament (p‐NF) immunohistochemistry reveals that in a control patient, the area corresponding to the olivospinal tract contains abundant small axon fibers among the large axon fibers. (D–F) An MSA patient with olivospinal tract degeneration (E arrowhead, F) displays loss of small axon fibers in the olivospinal tract, whereas large fibers have been relatively spared. (G–I) In contrast, an ALS patient demonstrates loss of large axon fibers in the anterior funiculi, while the small fibers appear to be spared. Scale bars: top row and second row, 200 μm; bottom row, 10 μm. ALS, amyotrophic lateral sclerosis; KB, Klüver‐Barrera staining; MSA, multiple system atrophy; p‐NF, phosphorylated neurofilament immunohistochemistry.

**FIGURE 4:**
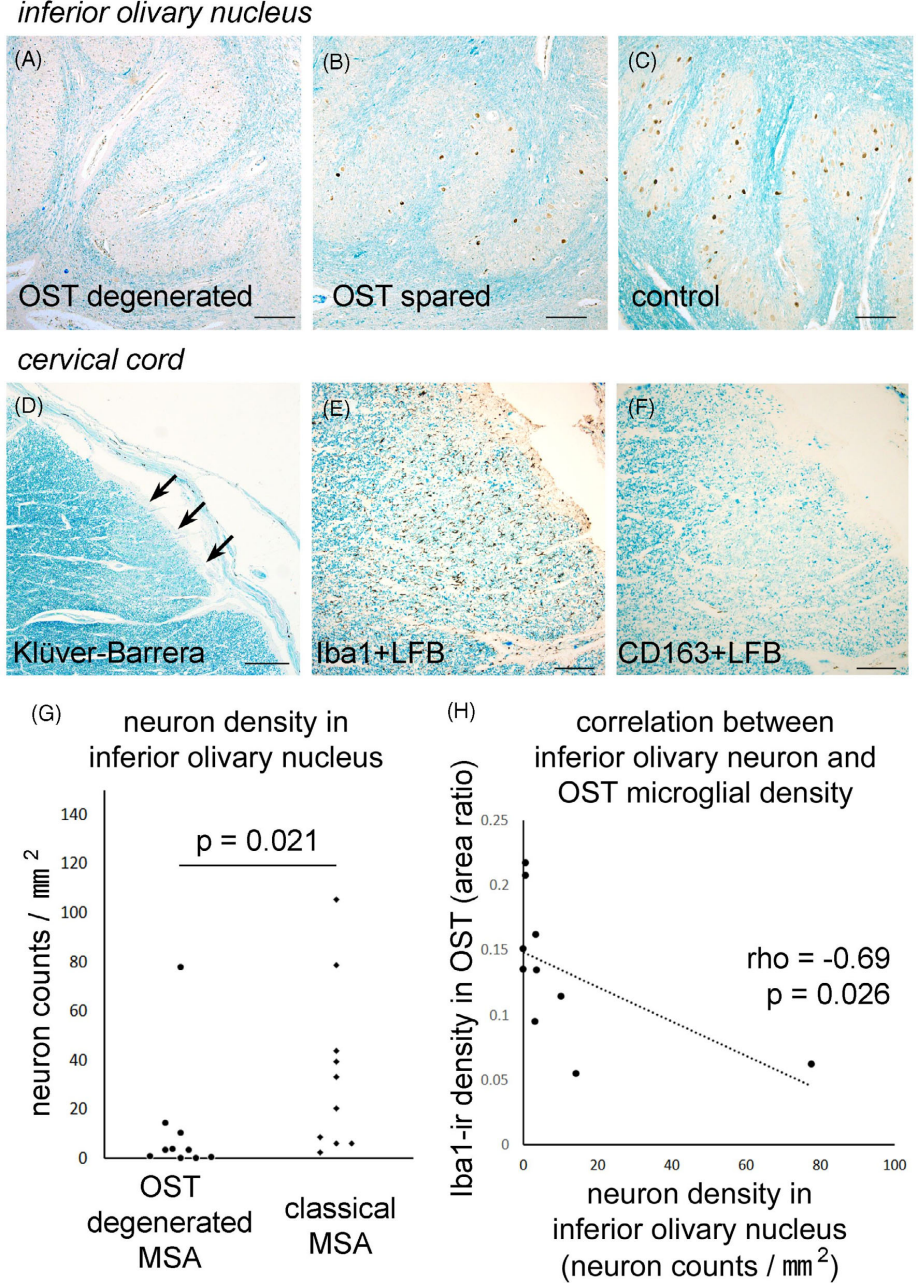
Correlation between olivospinal tract degeneration and neuronal loss in the inferior olivary nucleus. Anti‐NeuN immunohistochemistry, counterstained with luxol fast blue (LFB), revealed severe neuronal loss in the inferior olivary nucleus of a MSA patient with olivospinal tract (OST) degeneration (A) compared with a classical MSA patient (B) or a control patient (C). The degenerated olivospinal tract (D, arrows) showed severe microgliosis (E), but the macrophage response was sparse (F). Neuronal densities within the inferior olivary nucleus were significantly lower in MSA patients with olivospinal tract degeneration compared to those without degeneration (*p* = 0.021, Mann–Whitney *U*‐test) (G). In patients with olivospinal tract degeneration, microglial densities were negatively correlated with neuronal densities in the inferior olivary nucleus (rho = −0.69, *p* = 0.026. Spearman's rank correlation) (H). Scale bars: (A–C, E, F) 100 μm and (D) 500 μm. (A–C) Anti‐NeuN immunohistochemistry and LFB counterstaining, (D) Klüver‐Barrera method, (E) anti‐Iba1 immunohistochemistry and LFB counterstaining, and (F) anti‐CD163 immunohistochemistry and LFB counterstaining.

Table 1 shows the clinical findings of the enrolled patients. Twenty‐two (22.7%) out of 97 included patients with MSA showed bilateral triangular‐shaped tract degeneration in the boundary of the anterior and lateral funiculi. The tract degeneration appeared continuously across the cervical segments from C1 to C5 (Figures 1A and 2A–F). To identify the rostral and caudal ends of the tract degeneration, we obtained multiple 5‐mm thick slices from the pons to the cervical cord, which were then subjected to KB staining. This showed that the rostral end of the tract degeneration was nearby the inferior olivary nucleus at the bottom level of the medulla oblongata. The caudal end of the tract degeneration was consistently identified at the C5 level, and more caudal segments did not exhibit degeneration. The anatomical pathway of the degenerated tract was consistent with that of the olivospinal tract reported by Helweg [9]. Anti‐neurofilament immunohistochemistry revealed that the tracts within the olivospinal tract prominently consisted of small axon fibers (less than 2.5 μm in diameter) [23], in contrast with adjacent portions in the anterior and lateral funiculi primarily containing large axon fibers (Figure 3). Iba1‐immunopositive microglia were abundant within the olivospinal tract, whereas CD163‐immunopositive macrophages were sparse (Figure 4). Neurologically healthy controls and patients with LBD, ALS, PSP, SCA1, and SCA2 did not exhibit degeneration in the olivospinal tract. All ALS patients showed tract degeneration in the anterior funiculi to various extents, but large axon fibers were selectively depleted whereas small axon fibers were spared. This finding was in striking contrast with the degeneration of the olivospinal tract in MSA, which mostly involved small axon fibers (Figure 3).

**TABLE 1:** Clinical and neuropathological findings of the studied patients.

	MSA	Control	LBD	ALS	PSP	*p*
*N*	97	20	30	30	30	
Women/men	44/53	8/12	9/21	11/19	10/20	0.558^a^
Age at onset, years, mean ± SD (range)	60.6 ± 9.4 (42–82)	‐	68.4 ± 11.7 (40–89)	70.0 ± 11.1 (35–86)	72.1 ± 7.9 (56–86)	<0.0001^b^
Age at death, years, mean ± SD (range)	68.3 ± 7.9 (53–87)	67.8 ± 13.5 (31–83)	80.0 ± 7.4 (62–91)	74.0 ± 11.7 (54–88)	79.0 ± 6.4 (66–89)	<0.0001^b^
Olivospinal tract degeneration, *n* (%)	22 (22.7%)	0 (0%)	0 (0%)	0 (0%)	0 (0%)	<0.0001^a^
Brain weight, g, mean ± SD (range)	1171.0 ± 172.3 (700–1650) (*n* = 89)	1268.6 ± 190.2 (1030–1824) (*n* = 18)	1256.1 ± 139.4 (900–1550)	1258.5 ± 135.0 (1050–1570)	1170.7 ± 127.3 (936–1385) (*n* = 29)	0.00639^b^
Braak's NFT stage, mean ± SD (range)	1.4 ± 0.7 (1–4) (*n* = 96)	1.7 ± 0.8 (1–3)	2.8 ± 1.3 (1–6)	1.8 ± 0.8 (1–4)	2.1 ± 0.9 (1–4)	<0.0001^b^

Abbreviations: ALS, amyotrophic lateral sclerosis; LBD, Lewy body disease; MSA, multiple system atrophy; NFT, neurofibrillary tangle; PSP, progressive supranuclear palsy; SD, standard deviation.

^a^ Fisher's exact test.

^b^ Kruskal–Wallis test.

### 3.2 Clinical findings in MSA with olivospinal tract degeneration

We subdivided the 97 MSA patients into two groups: MSA patients with olivospinal tract degeneration (*n* = 22) and classical MSA patients without olivospinal tract degeneration (*n* = 75). Table 2 summarizes the clinical and neuropathological findings of these patients (raw data are presented in Tables S1 and S2). The age at disease onset was significantly lower in MSA patients with olivospinal tract degeneration (average: 56.4 ± 8.7 years, range: 42–74) compared to classical MSA patients (average: 61.8 ± 9.2 years, range: 43–82; Mann–Whitney *U* test, *p* = 0.0115). The disease duration was also significantly longer in MSA patients with olivospinal tract degeneration (average: 10.1 ± 4.8 years, range: 2–25) compared to classical MSA patients (6.8 ± 3.8 years, range: 1–19; Mann–Whitney *U* test, *p* = 0.000713). Three of the MSA patients with olivospinal tract degeneration (Patients 6, 10, 14 in Table S1) had undergone artificial ventilation 7, 10, and 11 years after disease onset, respectively. The distribution of sex and clinical phenotypes did not differ significantly between the two patient subgroups.

**TABLE 2:** Comparison between MSA with and without triangular tract degeneration.

	MSA with olivospinal tract degeneration	Classical MSA	*p*
*N*	22	75	
Women/men	10/12	34/41	1^b^
Age at onset, years, mean ± SD (range)	56.4 ± 8.7 (42–74)	61.8 ± 9.2 (43–82)	0.0115^c^
Duration of illness, years, mean ± SD (range)	10.1 ± 4.8 (2–25)	6.8 ± 3.8 (1–19)	0.000713^c^
Clinical phenotypes of MSA			
MSA‐C, *n* (%)	14 (63.6%)	40 (53.3%)	0.469^b^
MSA‐P, *n* (%)	7 (31.8%)	32 (42.7%)	0.461^b^
Unclassified^a^, *n* (%)	1 (4.5%)	3 (4.0%)	1^b^
Brain weight, g, mean ± SD (range)	1110 ± 226 (700–1110) (*n* = 19)	1187 ± 152 (700–1650) (*n* = 70)	0.155^c^
Jellinger's pathologic grade			
OPCA, mean ± SD (range)	2.7 ± 0.6 (1–3)	2.1 ± 0.9 (1–3)	0.00343^c^
SND, mean ± SD (range)	2.4 ± 0.8 (1–3)	2.3 ± 0.8 (1–3)	0.654^c^
Semi‐quantitative score of neuronal loss			
Pontine nucleus, mean ± SD (range)	1.9 ± 0.3 (1–2)	1.6 ± 0.5 (0–2)	0.0205^c^
Inferior olivary nucleus, mean ± SD (range)	1.8 ± 0.4 (1–2)	1.4 ± 0.7 (0–2)	0.0118^c^
Cerebellar cortex, mean ± SD (range)	2.0 ± 0.0	1.9 ± 0.2 (1–2)	0.277^c^
Lewy body in brainstem, *n* (%)	1 (4.5%)	4 (5.3%)	1^b^
Braak's NFT stage, mean ± SD (range)	1.4 ± 0.7 (1–3)	1.4 ± 0.7 (1–4) (*n* = 74)	0.774^c^
Thal's Aβ phase, mean ± SD (range)	0.7 ± 0.9 (0–3)	0.7 ± 1.0 (0–4) (*n* = 74)	0.962^c^

Abbreviations: MSA, multiple system atrophy; MSA‐C, MSA‐cerebellar type; MSA‐P, MSA‐parkinsonian type; NFT, neurofibrillary tangle; OPCA, olivopontocerebellar atrophy; SD, standard deviation; SND, striatonigral degeneration.

^a^ Unclassified MSA refers to patients who could not be classified as either MSA‐P or MSA‐C based on their clinical symptoms.

^b^ Fisher's exact test.

^c^ Mann–Whitney *U* test.

### 3.3 Olivospinal tract degeneration was associated with severe OPCA pathology

Jellinger's pathologic grades for OPCA were significantly more severe in MSA patients with olivospinal tract degeneration compared to classical MSA patients; 18 out of 22 MSA patients with olivospinal tract degeneration had grade III OPCA. In contrast, the severity of SND did not differ between the two subgroups (Figure 1B–G and Table 2). Semi‐quantitative assessment of each anatomical region revealed that neuronal loss in the pontine and inferior olivary nuclei was significantly more severe in MSA patients with olivospinal tract degeneration compared to those with classical MSA (Table 2).

### 3.4 Age‐related pathology

Age‐related or concomitant neuropathology, including Braak's NFT stages, Thal's beta‐amyloid phases, and the prevalence of Lewy bodies, did not differ between MSA patients with olivospinal tract degeneration and those with classical MSA (Table 2).

### 3.5 Relationship between olivospinal tract degeneration and neuronal loss in the inferior olivary nucleus

Quantitative analyses were performed in 10 MSA patients with olivospinal tract degeneration and 10 MSA patients without it. Disease duration and Jellinger's OPCA score were matched between the two subgroups (raw data are presented in Table S3). The analyses revealed that MSA patients with olivospinal tract degeneration had lower neuronal densities in the inferior olivary nucleus compared to those with classical MSA (Mann–Whitney *U*‐test, *p* = 0.021; Figure 4). We also assessed microglial density in the olivospinal tract from patients with MSA with olivospinal tract degeneration. There was a moderate, negative correlation between neuronal density in the inferior olivary nucleus and microglial density in the olivospinal tract (Spearman's rank correlation, rho = −0.69, *p* = 0.026; Figure 4).

### 3.6 GCI densities in the inferior olivary nucleus and olivospinal tract were correlated

**FIGURE 5:**
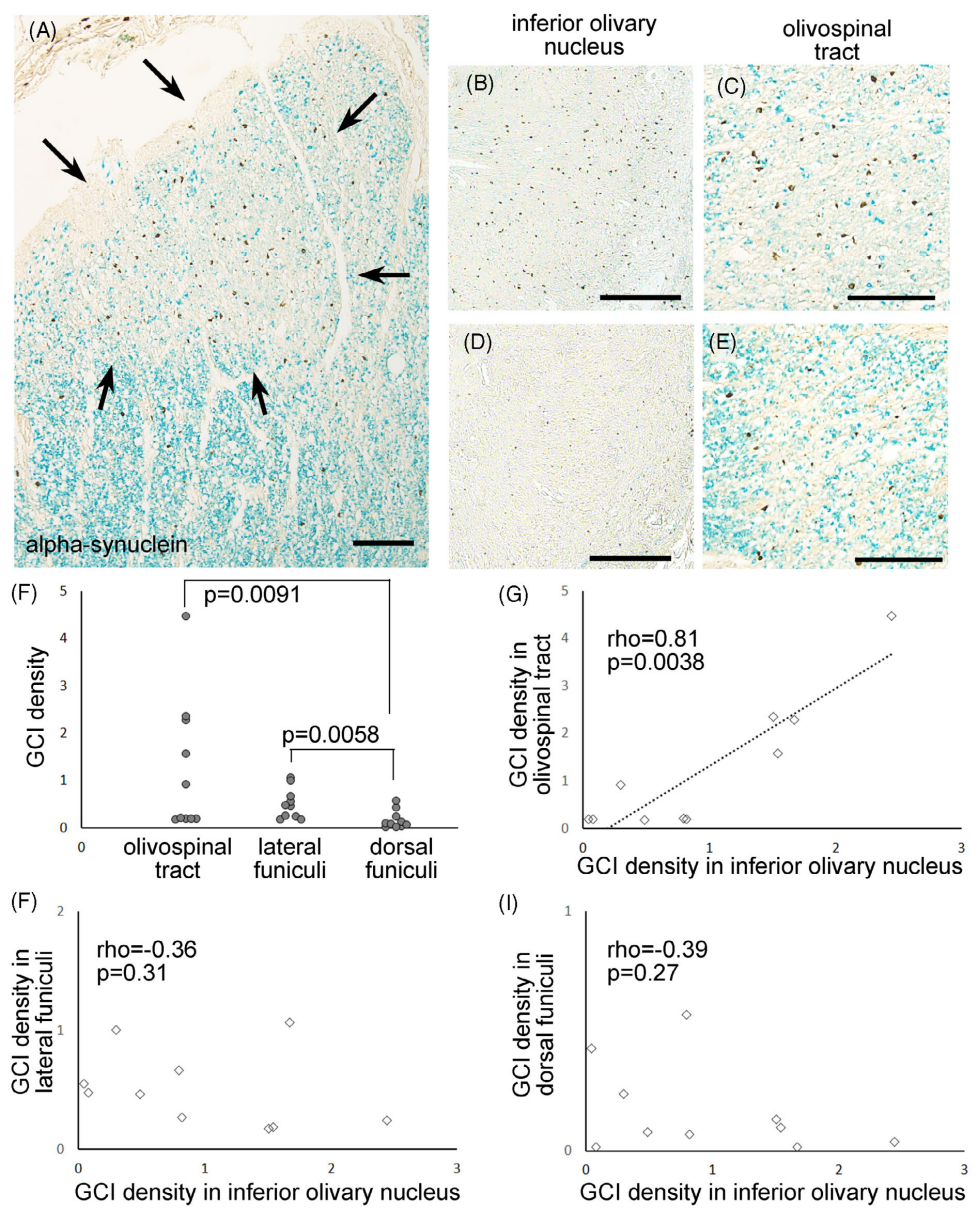
GCI density correlation between the inferior olivary nucleus and the olivospinal tract. (A) Alpha‐synuclein‐immunopositive GCIs were observed in the degenerated olivospinal tract (arrows). Note that GCIs were abundant in the olivospinal tract compared to adjacent regions of the anterior funiculi. (B, C) A patient with abundant GCIs in the inferior olivary nucleus (B) had abundant GCIs in the olivospinal tract as well (C). (D, E) In contrast, another patient with sparse GCIs in the inferior olivary nucleus (D) exhibited few GCIs in the olivospinal tract (E). (F) GCI density in the olivospinal tract or the lateral funiculi was significantly higher than that in the dorsal funiculi (Mann–Whitney *U*‐test, *p* = 0.0091 and 0.0058, respectively. Significance levels were set at 0.016 after Bonferroni correction). No correlation was observed between GCI densities in the olivospinal tract and the lateral funiculi (*p* = 0.62). (G–I) GCI density in the inferior olivary nucleus was positively correlated with that in the olivospinal tract (rho = 0.81, *p* = 0.0038, Spearman's rank correlation) but not with that in the lateral and dorsal funiculi. Scale bars: (A, C, and E) 100 μm, (B and D) 200 μm. (A–E) Anti‐alpha‐synuclein immunohistochemistry with LFB counterstaining.

We assessed densities of alpha‐synuclein‐immunopositive GCIs in the inferior olivary nucleus and olivospinal tract in the 10 MSA patients with olivospinal tract degeneration. The assay revealed that GCI density within the olivospinal tract was positively and strongly correlated with that within the inferior olivary nucleus (Spearman's rank correlation, rho = 0.81, *p* = 0.038; Figure 5).

### 3.7 Olivospinal tract degeneration was independent from degeneration of the dorsal and lateral funiculi

A subset of MSA patients showed tract degeneration in the dorsal or lateral funiculi (12 in the dorsal funiculi and 23 in the lateral funiculi, respectively). However, the prevalence of tract degeneration in these portions did not significantly differ between MSA patients with and without olivospinal tract degeneration (dorsal column: *p* = 0.460, Fisher's exact test, 4 and 8 out of the MSA patients with olivospinal tract degeneration and those without, respectively; corticospinal tract: *p* = 0.393, 7 and 16 out of the MSA patients with olivospinal tract degeneration and those without, respectively).

## 4 DISCUSSION

To the best of our knowledge, this study is the first to report degeneration of the olivospinal tract in patients with MSA. The degenerated tract mostly comprised small axon fibers, and is located in the boundary of the anterior and lateral funiculi, running across the medullary bottom and C5 segments. These anatomical features are consistent with the original description of the olivospinal tract by Helweg [9]. Quantitative results revealed that olivospinal tract degeneration is linked to severe neuronal loss in the inferior olivary nucleus, and that the reduction of olivary neurons is correlated with microgliosis in the olivospinal tract. We also revealed that GCI densities in the inferior olivary nucleus and olivospinal tract are strongly correlated. The study results indicate an impairment of neural connectivity between the inferior olivary nucleus and the spinal cord in MSA, and the pathological involvement of the olivospinal tract may develop in a descending manner. Our observations also revealed that the olivospinal tract degeneration appears to be independent from the degeneration of the lateral funiculi or dorsal funiculi, suggesting that it is not explained by the degeneration of other ascending or descending tracts.

Clinicopathological analyses showed that MSA patients with olivospinal tract degeneration had lower ages at disease onset, longer clinical durations, and more severe OPCA pathology compared to classical MSA patients. These findings suggest the possibility that olivospinal tract degeneration may occur during advanced disease after a long course of OPCA. Indeed, histopathological observations showed severe microgliosis in the olivospinal tract, whereas macrophage response was almost absent.

According to our observations, olivospinal tract degeneration is characteristic of MSA patients. Neurologically healthy controls, or patients with LBD, ALS, PSP, SCA1, or SCA2 did not show this change. It is well known that the anterior funiculi, as well as the lateral funiculi, are often involved in ALS [24]. Hence, whether degeneration of the anterolateral funiculi in ALS can be distinguished from olivospinal tract degeneration in MSA should be determined. Our study showed that large axons were prominently depleted in the anterior as well as in the lateral funiculi of ALS patients. This was in striking contrast with olivospinal tract degeneration in MSA patients, which was characterized by prominent involvement of small axon fibers. Moreover, none of the ALS patients showed a well‐circumscribed degeneration of the olivospinal tract. In ALS, tract degeneration within the anterior funiculi may primarily arise from the involvement of the anterior corticospinal tract. Hence, degeneration of the anterolateral funiculi in ALS patients is clearly different from that occurring in the olivospinal tract of MSA patients.

There have been some controversies regarding the first report by Helweg on the olivospinal tract [10, 11, 12]. He identified a triangular tract comprising small axons in the boundary between the anterior and lateral funiculi across C1 and C5 segments on autopsied psychiatric patients in 1888 [9]. He made serial sections ranging from the pons to the cervical cord using carmine as staining agent, and concluded that this tract was a descending olivospinal tract. Studies on animals, however, suggested that Helweg's triangular tract was ascending spino‐olivary tract [25], possibly originating from posterior horn neurons of lumbrosacral segments and intermediate zone neurons at all segments [11]. Another study reported that olivary atrophy did not affect Helweg's triangular tract, and suggested origins other than the inferior olivary nucleus for this tract, although this was based on an observation from a single male patient with olivary atrophy of unknown etiology [10]. In contrast, a later study on human tissue revealed Helweg’ triangular tract to be a descending tract by detailed observation of celloidin‐embedded multiple sections [23]. Although our study does not preclude the existence of an ascending spino‐olivary tract, our finding from the observed MSA patients implies olivospinal tract degeneration in a descending manner. If the patients had shown degeneration of the ascending spino‐olivary tract originating from lumbrosacral posterior horn neurons, degeneration of the dorsal funiculi would be associated to it. Importantly, our observations did not show any correlation between the degeneration of these two tracts. In addition, a dying‐back process of the ascending spino‐olivary tract from neuronal loss in the inferior olivary nucleus seems unlikely. GCI densities in the inferior olivary nucleus and the degenerated tract were strongly correlated, indicating that tract degeneration is primarily a consequence of alpha‐synucleinopathy in the inferior olivary nucleus, not a secondary process. Moreover, the tract degeneration was consistently observed from C1 to C5 in our MSA patients. It is hard to explain why the dying‐back process always ceased at the C5 level in the patients.

The present study had several limitations. First, magnetic resonance imaging (MRI) of the spinal cord from the patients included in the study was not available. Hence, the usefulness of olivospinal tract degeneration as a radiological marker of MSA remains unclear. Second, the clinical findings were not evaluated consistently or longitudinally using standardized clinical scales due to the retrospective design of the study. The physiological functions of the olivospinal tract are also unknown. Although we cannot precisely identify the clinical impact of olivospinal tract degeneration for MSA patients, prospective clinical assessments together with spinal cord MRI might address this problem.

In conclusion, we observed olivospinal tract degeneration in autopsied MSA patients. The tract degeneration was closely linked to neuronal loss in the inferior olivary nucleus and an OPCA phenotype associated with long clinical duration. Although the physiological impact of this change remains unclear, an impairment of the neural connection between the inferior olivary nucleus and spinal cord is implied.

## AUTHOR CONTRIBUTIONS

**Takashi Ando**: Conceptualization, methodology, investigation, writing—original draft. **Yuichi Riku**: Methodology, investigation, writing—review & editing. **Akio Akagi**: Investigation. **Hiroaki Miyahara**: Investigation. **Takashi Uematsu**: Investigation. **Ikuko Aiba**: Investigation. **Jun Sone**: Investigation. **Masahisa Katsuno**: Writing—review & editing, supervision. **Mari Yoshida**: Conceptualization, methodology, investigation, writing—review & editing, supervision. **Yasushi Iwasaki**: Investigation, writing—review & editing, supervision.

## FUNDING INFORMATION

M.Y. received an Intramural Research Grant (30–8) for Neurological and Psychiatric Disorders from the NCNP. Y.R. received a grant from JSPS KAKENHI (JP23K06935) and the HORI Science and Arts Foundation. Y.I. received Health, Labor, and Welfare Sciences Research Grants and Grants‐in‐Aid from the Research Committee of CNS Degenerative Diseases, Research on Policy Planning and Evaluation for Rare and Intractable Diseases, and the Ministry of Health, Labor, and Welfare, Japan. This research was partly supported by AMED under Grant Number JP22wm0425019 (M.Y.) and the Japanese Red Cross Aichi Medical Center Nagoya Daiichi Hospital Research Grant NFRCH 23‐0002 (T.A.).

## CONFLICT OF INTEREST STATEMENT

The authors report no competing interests.

## ETHICS STATEMENT

Written informed consent was obtained from the patients' relatives before autopsies. All human studies were approved by the research ethical committee of Aichi Medical University.

## Supporting information


**Figure S1.** Quantitative assessments.
**Table S1.** Clinical and neuropathological findings in MSA with triangular tract degeneration.
**Table S2.** Semi‐quantitative scores of neuronal loss in MSA with triangular tract degeneration.
**Table S3.** Demographic data from patients subjected to quantitative assessment.

## Data Availability

The raw data that support the findings of this study are available upon reasonable request from the corresponding author.
