# Supplementary material for: Degeneration of olivospinal tract in the upper cervical spinal cord of multiple system atrophy patients: Reappraisal of Helweg's triangular tract
Source: Brain Pathol. 2023 Nov 16;34(3):e13226. doi: 10.1111/bpa.13226 (PMC11007009; doi:10.1111/bpa.13226)
Supplement: Supplementary file 1 — Figure S1. Quantitative assessments. Table S1. Clinical and neuropathological findings in MSA with triangular tract degeneration. Table S2. Semi‐quantitative scores of neuronal loss in MSA with triangular tract degeneration. Table S3. Demographic data from patients subjected to quantitative assessment. [file BPA-34-e13226-s001.docx]

**
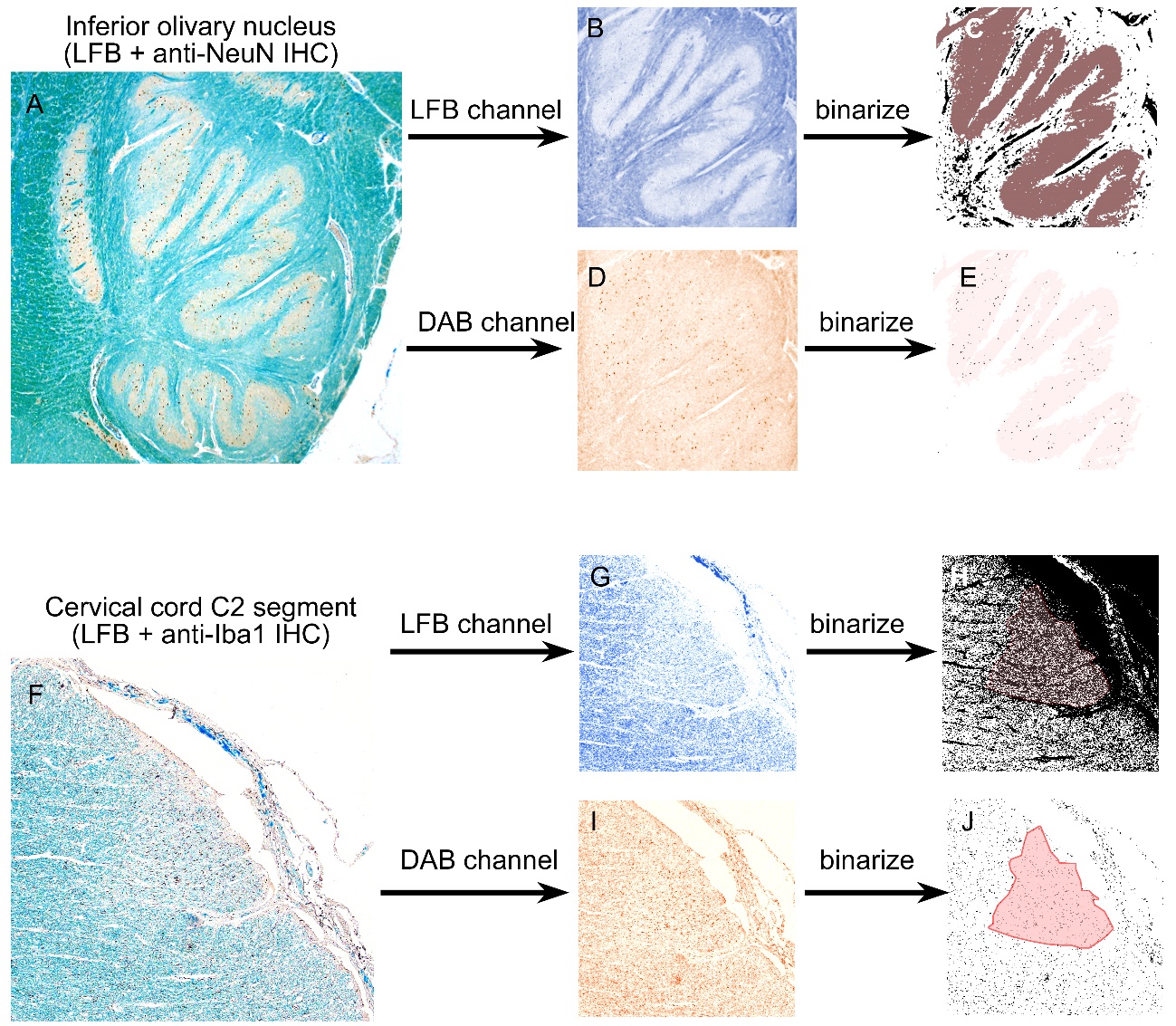
**

**Figure S1. Quantitative assessments.**

To assess neuronal density in the inferior olivary nucleus, we prepared slides of medulla oblongata sliced at 5 mm caudal from the obex. The slides underwent anti-NeuN immunohistochemistry and with luxol fast blue (LFB) staining (A). Photomicrographs of the inferior olivary nucleus were captured, and the ‘color-deconvolution’ plug-in of Image-J allowed us to automatically split the LFB and DAB channels (B-E). Binarized images were generated using the ‘Otsu threshold’ of Image-J under the same settings (C and E). Then, the inferior olivary nucleus and individual neurons were automatically selected by ‘magic wand’ and ‘particle counts’ operations of Image-J, respectively. It is known that anti-NeuN immunohistochemistry recognizes not only neuronal nucleus but also neuronal soma and processes. Hence, we only counted NeuN-immunopositive particles with more than 7 μm in diameter to avoid multiple counts of neuronal processes from a single neuron. Assessment of microglia in the olivospinal tract was performed using a similar method. We prepared slices of the C2 segment from MSA patients with olivospinal tract degeneration. Anti-Iba1 immunohistochemistry and LFB staining were performed (F). The photomicrographs underwent splitting of the LFB and DAB channels (G-J), followed by binarization. Image capture and computation for the medulla oblongata and cervical cord were performed bilaterally, and an average of the values obtained for each side was recorded as the outcome of each slide. Densities of GCIs in the olivospinal tract and the lateral funiculi (averaged from both sides), and the dorsal funiculi were calculated via anti-alpha-synuclein immunohistochemistry with LFB counterstaining.

**Table S1. Clinical and neuropathological findings in MSA with triangular tract degeneration**

| Patient | Sex | Age at onset, years | Duration of illness, years | Initial symptoms | Clinical phenotype | Brain weight, grams | Jellinger’s pathologic grade | | Lewy bodies in brainstem | Braak’s NFT stage |
| --- | --- | --- | --- | --- | --- | --- | --- | --- | --- | --- |
|  |  |  |  |  |  |  | OPCA | SND |  |  |
| 1 | F | 74 | 2 | Postprandial hypotension | P | 1,120 | II | III | - | III |
| 2 | F | 51 | 3 | Voiding difficulties | C | NA | III | II | - | I |
| 3 | M | 55 | 5 | Gait difficulty | C | 1,430 | III | II | - | I |
| 4 | M | 73 | 6 | Gait difficulty | C | 1,160 | II | I | - | II |
| 5 | M | 51 | 6 | Voiding difficulties | C | 1,250 | I | II | - | I |
| 6 | F | 59 | 7 | Gait difficulty | P | 1,080 | III | III | - | II |
| 7 | M | 55 | 8 | Dizziness | C | NA | III | I | - | I |
| 8 | M | 62 | 8 | Voiding difficulties | P | 1,260 | I | III | - | I |
| 9 | M | 59 | 9 | Gait difficulty | C | 1,234 | III | I | - | II |
| 10 | M | 42 | 10 | Gait difficulty | C | NA | III | III | - | I |
| 11 | M | 54 | 10 | Gait difficulty | C | 1,590 | III | I | - | I |
| 12 | M | 52 | 11 | Gait difficulty | C | 1,100 | III | II | - | I |
| 13 | M | 50 | 11 | Hand clumsiness | P | 1,260 | III | III | - | I |
| 14 | F | 53 | 11 | Gait difficulty | C | 1,050 | III | II | - | I |
| 15 | F | 53 | 12 | Bradykinesia | P | 810 | III | III | - | II |
| 16 | F | 73 | 12 | Gait difficulty | C | 805 | III | III | - | I |
| 17 | M | 50 | 13 | Gait difficulty | C | 1,340 | III | II | - | I |
| 18 | F | 49 | 13 | Gait difficulty | C | 940 | III | III | - | I |
| 19 | M | 65 | 13 | Gait difficulty | C | 900 | III | III | - | I |
| 20 | F | 55 | 13 | Gait difficulty | U | 960 | III | III | - | I |
| 21 | F | 60 | 14 | Bradykinesia | P | 1,110 | III | III | + | III |
| 22 | F | 45 | 25 | Falls | P | 700 | III | III | - | I |

Abbreviations: C, multiple system atrophy-cerebellar type; F, female; M, male; MSA, multiple system atrophy; NA, not available; NFT, neurofibrillary tangle; OPCA, olivopontocerebellar atrophy; P, multiple system atrophy-parkinsonian type; SND, striatonigral degeneration; U, unclassified

**Table S2. Semi-quantitative scores of neuronal loss in MSA with triangular tract degeneration**

| Patient | Pontine base | Inferior olivary nucleus | Cerebellar cortex | Putamen | Substantia nigra | Prefrontal cortices | Precentral cortices |
| --- | --- | --- | --- | --- | --- | --- | --- |
| 1 | 2+ | 1+ | 2+ | 2+ | 2+ | 0 | 0 |
| 2 | 2+ | 2+ | 2+ | 2+ | NE | NE | NE |
| 3 | 2+ | 2+ | 2+ | 2+ | 2+ | 0 | 0 |
| 4 | 2+ | 1+ | 2+ | 1+ | 2+ | 0 | 0 |
| 5 | 1+ | 1+ | 2+ | 2+ | 1+ | 0 | 1+ |
| 6 | 2+ | 2+ | 2+ | 2+ | 2+ | 0 | 1+ |
| 7 | 2+ | 2+ | 2+ | 1+ | 2+ | 0 | 0 |
| 8 | 1+ | 1+ | 2+ | 2+ | 2+ | 0 | 0 |
| 9 | 2+ | 2+ | 2+ | 1+ | 2+ | 1+ | 1+ |
| 10 | 2+ | 2+ | 2+ | 2+ | 2+ | 0 | 0 |
| 11 | 2+ | 2+ | 2+ | 1+ | 2+ | 0 | 1+ |
| 12 | 2+ | 2+ | 2+ | 2+ | 2+ | 0 | 0 |
| 13 | 2+ | 2+ | 2+ | 2+ | 2+ | 0 | 1+ |
| 14 | 2+ | 2+ | 2+ | 2+ | 2+ | 1+ | 1+ |
| 15 | 2+ | 2+ | 2+ | 2+ | 2+ | 1+ | 1+ |
| 16 | 2+ | 2+ | 2+ | 2+ | 2+ | 0 | 1+ |
| 17 | 2+ | 2+ | 2+ | 2+ | 2+ | 1+ | 1+ |
| 18 | 2+ | 2+ | 2+ | 2+ | 2+ | 0 | 1+ |
| 19 | 2+ | 2+ | 2+ | 2+ | 2+ | 0 | 1+ |
| 20 | 2+ | 2+ | 2+ | 2+ | 2+ | 1+ | 1+ |
| 21 | 2+ | 2+ | 2+ | 2+ | 2+ | 1+ | 1+ |
| 22 | 2+ | 2+ | 2+ | 2+ | 2+ | 1+ | 1+ |

Abbreviations: NE, not evaluated due to coexisting mild ischemic changes

Semi-quantitative assessment: grade 0, normal appearance; grade 1+, definite astrogliosis in the absence of definite neuronal loss; grade 2+, apparent neuronal loss and astrogliosis

**Table S3. Demographic data from patients subjected to quantitative assessment**

Abbreviations: MSA, multiple system atrophy; OPCA, olivopontocerebellar atrophy; SND, striatonigral degeneration
